# Blind search for post-translational modifications and amino acid substitutions using peptide mass fingerprints from two proteases

**DOI:** 10.1186/1756-0500-1-130

**Published:** 2008-12-19

**Authors:** Harald Barsnes, Svein-Ole Mikalsen, Ingvar Eidhammer

**Affiliations:** 1 Department of Informatics, University of Bergen, PB 7803, N-5020 Bergen, Norway; 2 Institute for Cancer Research, The Radium Hospital, The National Hospital Medical Center, Montebello, N-0310 Oslo, Norway

## Abstract

**Background:**

Mass spectrometric analysis of peptides is an essential part of protein identification and characterization, the latter meaning the identification of modifications and amino acid substitutions. There are two main approaches for characterization: (i) using a predefined set of possible modifications and substitutions or (ii) performing a blind search. The first option is straightforward, but can not detect modifications or substitutions outside the predefined set. A blind search does not have this limitation, and therefore has the potential of detecting both known and unknown modifications and substitutions. Combining the peptide mass fingerprints from two proteases result in overlapping sequence coverage of the protein, thereby offering alternative views of the protein and a novel way of indicating post-translational modifications and amino acid substitutions.

**Results:**

We have developed an algorithm and a software tool, MassShiftFinder, that performs a blind search using peptide mass fingerprints from two proteases with different cleavage specificities. The algorithm is based on equal mass shifts for overlapping peptides from the two proteases used, and can indicate both post-translational modifications and amino acid substitutions. In most cases it is possible to suggest a restricted area within the overlapping peptides where the mass shift can occur. The program is available at .

**Conclusion:**

Without any prior assumptions on their presence the described algorithm is able to indicate post-translational modifications or amino acid substitutions in MALDI-TOF experiments on identified proteins, and can thereby direct the involved peptides to subsequent TOF-TOF analysis. The algorithm is designed for detailed and low-throughput characterization of single proteins.

## Background

The detection and verification of post-translational modifications in proteins and peptides by mass spectrometry (MS) is a common technique in protein characterization. The protein is proteolytically cleaved into peptides and analyzed by MS. MALDI-TOF instruments generate a list of mass-over-charge ratios (m/z values), referred to as a peptide mass fingerprint (PMF), which is compared to theoretical PMFs of known proteins. Modifications can be included in the theoretical PMFs. However, including too few can result in undetected modifications, while selecting too many can result in wrongly suggested modifications. One option is to perform the search in two iterations, where first a few expected modifications are considered. Thereafter, unmatched peptides are submitted to a modification search, e.g., in FindMod [1] or MassSorter [2]. FindMod only considers 22 common modifications. Here we present an alternative approach using blind search, where PMF data from two proteases on two aliquots of a sample are used to indicate modifications and amino acid substitutions. If the same mass shift relative to the unmodified theoretical values is observed for both proteases, and the peptides are overlapping, the mass shift can correspond to a modification or a substitution. MacCoss et al. [3] used a similar reasoning, but only to verify a limited set of predefined modifications in LC-MS/MS experiments. Unrestricted search for modifications using LC-MS/MS data has been developed more recently [4,5].

**Figure 1:**
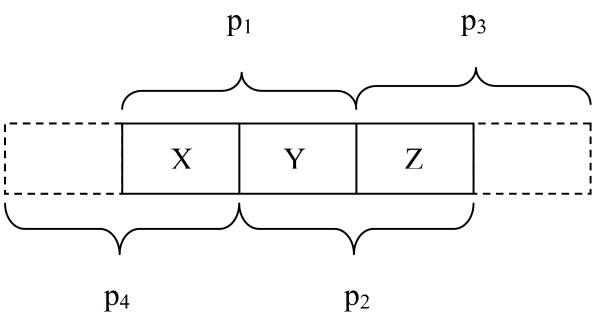
**Overlapping peptides**. The peptides p_1 _and p_2 _define different regions (*X*, *Y*, *Z*) of the covered area as explained in the text. The figure also indicates that if adjacent peptides p_3 _and p_4 _are found, they can be combined with p_2 _and p_1_, respectively, to strengthen the probability for found mass shifts in X or Z being real mass shifts.

Figure 1 shows two overlapping peptides, p_1 _and p_2_, generated by different proteases. Let p_1 _be the most N-terminal peptide, and p_2_ the most C-terminal peptide. The overlapping peptides define three areas: the overlapping area, *Y*; the area of p_1 _not overlapping with p_2_, *X *(N-terminal area); and the area of p_2 _not overlapping with p_1_, *Z *(C-terminal area). Together, these will be referred to as the covered area. Note that p_1 _and p_2 _may have the same start or end residue, or that one peptide can completely cover the other. The main idea for our method is that a modification or an amino acid substitution occurring in area *Y *can be detected as an equal mass shift in p_1 _and p_2_. Equal mass shifts occurring in *X *and *Z*, but not in *Y*, can also be detected. This means that the non-overlapping areas *X *and *Z *both contain the same modified amino acid, or different amino acids carrying an identical modification, e.g., phosphorylation on S and T.

Let (i) p_1 _and p_2 _be two overlapping theoretical peptides; (ii) t_1 _be the theoretical mass of p_1_, and t_2 _be the theoretical mass of p_2_; and (iii) e_1 _be an experimental mass using protease A, and e_2 _be an experimental mass using protease B. Suppose the following equation is observed: e_1 _- t_1 _= e_2 _- t_2 _= Δm. Δm is then either a real mass shift or an artifact.

*Real mass shifts*: e_1 _corresponds to p_1_, and e_2 _corresponds to p_2_. The mass shift can then (i) occur solely in *Y*; (ii) occur in both *X *and *Z*; or (iii) the mass shifts occur as a combination of the two former cases. In the first case, the mass shift can correspond to one or more modifications/substitutions, while in the other cases, two or more modifications are needed.

*Artifact*: at least one of the masses e_1 _or e_2 _does not correspond to p_1 _or p_2 _respectively. Artifacts are covered in more detail in the Discussion.

## Findings

### Algorithm

The following algorithm detects equal mass shifts in overlapping peptides:

1. Let E_1 _be the peptide mass list from an experiment using protease A, and E_2 _be the peptide mass list from an experiments using protease B.

2. Let T_1 _be the list of theoretical peptide masses resulting from an in silico digestion using protease A, and T_2 _be the list of theoretical peptide masses resulting from an in silico digestion using protease B.

3. Remove from E_1 _all peaks corresponding to unmodified peptides in T_1 _and all peaks corresponding to autolytic peaks from protease A.

4. Repeat Step 3 with mass lists E_2 _and T_2 _from protease B.

5. Compare each mass e_i _∈ E_1 _to each mass t_j _∈ T_1_, and each mass e_k _∈ E_2 _to each mass t_m _∈ T_2_. Store the mass shifts (e_i _- t_j_) for all *i *and *j *and the mass shifts (e_k _- t_m_) for all *k *and *m*, in two lists M_1 _and M_2_, which now contain all possible mass shifts between corresponding experimental and theoretical data.

6. Let p_j _and p_m _be the theoretical peptides corresponding to t_j _and t_m _respectively. Compare M_1 _and M_2 _and find all pairs such that:

a. |(e_i _- t_j_) - (e_k _- t_m_)| = ω and (ω is the mass shift accuracy)

b. |(e_i _- t_j_)| > ε and |(e_k _- t_m_)| > ε and (ε is the mass shift threshold)

c. p_j _and p_m _overlap

The output is a list of overlapping peptides from E_1 _and E_2 _with equal mass shifts. The reason for the mass shifts, i.e., modification(s) or substitution(s), has to be positioned in the covered area, and have a mass equal to the detected mass shift. The list should be cross-checked against a database of known modifications and substitutions (e.g., UniMod [6,7]), and/or the included peptides can be tested in additional experiments, i.e., by MALDI-TOF-TOF, verifying or rejecting the proposed modification or substitution.

### Implementation

The described algorithm is implemented in Java [8] and available as a software tool, MassShiftFinder, at .

The main input to MassShiftFinder is the protein sequence and the experimental masses from two PMF experiments on the same protein using different proteases. Before running the algorithm it is recommended to remove all identified peptides from the PMFs, e.g., by using MassSorter [2]. Unmodified peptides, autolytic protease peaks and known noise/contaminating peaks (e.g., keratin) can be filtered within adjustable accuracy limits in the program. Using filters limits the number of unnecessary mass shift comparisons (see additional file 1, Fig. 1 (TheoreticalExamples.pdf)).

In order to reduce search space and increase the possibility of detecting real mass shifts, the following parameters should be set to reasonable values. (i) Mass Shift Threshold, where mass shifts below this threshold are excluded to avoid spurious comparisons among very small mass shifts. We would in general recommend setting this value to 0.9 to achieve the inclusion of deamidations. (ii) Mass Shift Boundaries, determine the search limits for a mass shift being a modification or substitution. It can be set to a more limited mass range, e.g., 79–81 Da to search for phosphorylations. (iii) Mass Shift Accuracy, where equal mass shifts are recognized when the difference between two mass shifts are within this accuracy (in Da or ppm). We would in general recommend setting this parameter at 0.2 Da when 25 ppm accuracy limit is used for the experimental peptides, and to decrease it if the instrument is more exact. Note that this parameter refers to inaccuracy of the potential modification as calculated from the comparison of experimental data and the theoretical peptide sequence.

**Figure 2:**
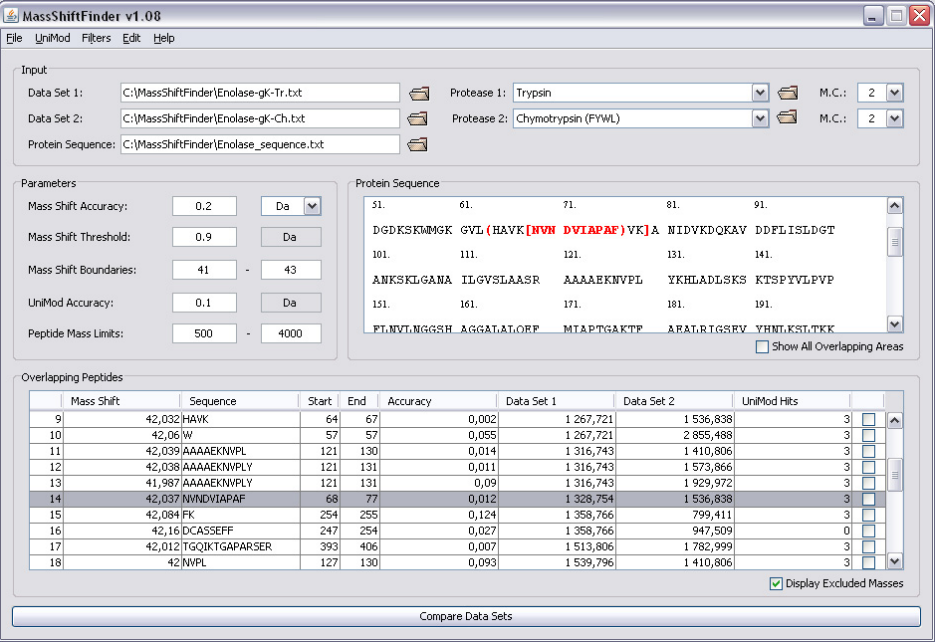
**Screenshot of the MassShiftFinder main window**. The data are taken from an experiment on guanidinated enolase (see additional file 2: ExperimentalExamples.pdf). The mass shift boundaries are set to restrict the detected mass shift mainly to guanidinations (mass shift of 42 Da). The highlighted row (14) indicates an "*X*-*Z*" mass shift (see Figure 1). Rows 11–13 show a mass shift occurring in the *Y*-area, with one tryptic peptide (1316.7) paring up with three chymotryptic peptides. The right-most column (UniMod hits) for most of the rows indicates that there are three modifications (acetylation, tri-methylation and guanidination) that are consistent with a mass shift of 42 for the indicated peptides. Row 16 has 0 UniMod hits because the calculated mass shift is more than 0.1 Da from the three mentioned modifications; furthermore, this mass shift cannot be due to guanidinated K as only one of the peptides contains a K.

An example of output is shown in Figure 2. By selecting a row, the overlapping peptides are indicated in the protein sequence. The detected mass shifts are searched against a local version of the UniMod database. To reduce the amount of incorrect UniMod explanations, this search can be restricted by choosing the allowed modification types, e.g., amino acid substitutions, post-translational modifications, etc. Up to two modifications per peptide are supported. Note that changing the settings for the UniMod search only affects the number of suggested explanations for each mass shift, not the number of mass shifts. Unexplained mass shifts may correspond to unknown modifications or more than two modifications per peptide. An example showing detection of modifications in an artificial dataset is found in additional file 1 (TheoreticalExamples.pdf).

### Experimental Example

We compared connexin43 (Cx43) [9] from three species. The experimental peak lists of Cx43 from Syrian hamster, Chinese hamster and rat were collected in MassSorter [2] using the Syrian hamster sequence as basis of comparison [10]. After removing autolytic protease peaks, peaks from the contaminating antibody and peaks in common with Syrian hamster, the remaining peaks were inserted into MassShiftFinder using the following parameters: Filter Accuracy and Unmodified Peptide Accuracy, 50 ppm (found under Edit/Preferences); Mass Shift Accuracy, 0.2 Da; Mass Shift Threshold, 0.9 Da; Mass Shift Boundaries, -200 to 200 Da; UniMod Accuracy, 0.1 Da; Missed Cleavages, 1; and including only amino acid substitutions in the search.

**Figure 3:**
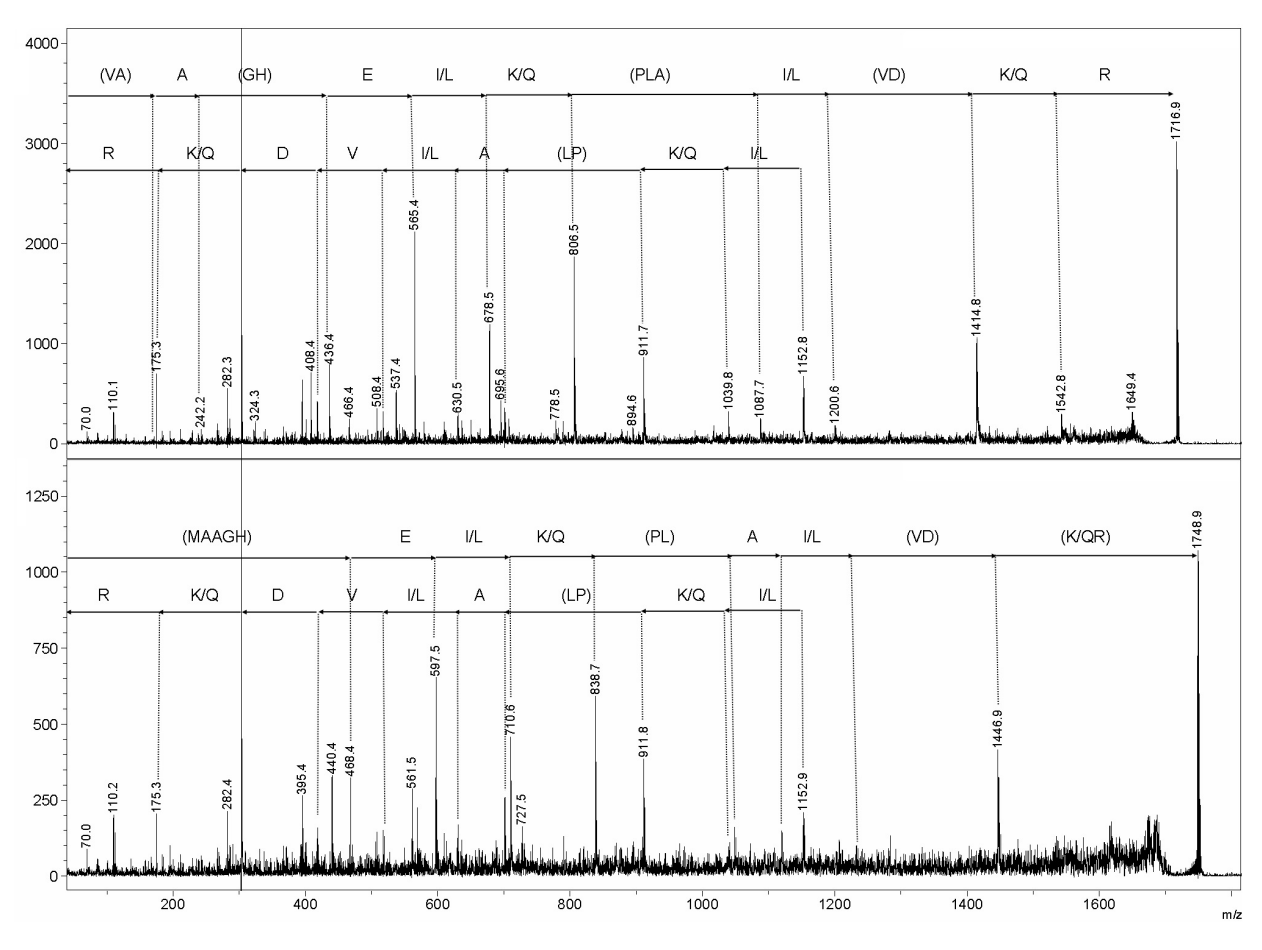
**TOF-TOF data of the Cx43 peaks at m/z 1716.84 and m/z 1748.91**. The peptides 1716.84 (upper) and 1748.91 (lower) are from rat and Chinese hamster Cx43, respectively. Note that the y_2_-ion at m/z 303.2 had higher intensity than all other ions. In both panels, the upper sequence is read from the b-ions, and the lower sequence is read from the y-ions. Further note that also the a_5_- to a_8_-ions can be distinguished in the upper panel, and the a_5_- and a_6_-ions in the lower panel. See text for more explanation.

For Chinese hamster, MassShiftFinder pointed out a potential substitution within the area 347-IAAGHELQPL-356 with a mass shift of 17.96 Da. This would correspond to a substitution from I or L to M. The rat data also indicated a potential substitution in the same sequence with a mass shift of -14.02 Da. This could correspond to a substitution from A to G, E to D, or I or L to V. The Chinese hamster and rat peptides with m/z 1748.91 and m/z 1716.84 (corresponding to mass shifts of 17.95 Da and -14.02 Da relative to the Syrian hamster peptide with m/z 1730.96) were targeted for TOF-TOF analysis (Fig. 3). The only possible substitution in Chinese hamster that is consistent with all data is a change in position 347 from I (Syrian hamster) to M (Chinese hamster). For rat, both I347 to V and A348 to G are consistent with these data. The former is the correct alternative. This example shows that our approach can be used to narrow the range of possibilities when detecting amino acid substitutions. For more examples and details, see additional file 2 (ExperimentalExamples.pdf).

## Discussion

The algorithm depends on good experimental sequence coverage and overlapping peptides. Sequence coverage mainly depends on the amino acid sequence, the sample amount, the protease used, and purity. An analysis of human proteins in SwissProt suggests that approximately 70–90% of the proteins have a theoretical coverage between 50 and 100%, regardless of whether trypsin, chymotrypsin or gluC was used (see additional file 3: SupplementaryMaterials.pdf). The experimental sequence coverage is usually lower than the theoretical upper limit, but a considerable degree of experimental overlap would generally be expected.

A detected mass shift (Δm) can either be real, i.e., resulting from a modification/substitution, or an artifact. Although unknown modifications still can be found [4], it is more likely that a mass shift is due to a known modification. Following the parsimony principle, it seems reasonable to first assume that a mass shift is caused by a single known modification. Accepted modified peptides can then be removed before subsequent searches are performed with less restricted parameters, e.g., allowing two modifications per peptide.

The tendency for artifacts is augmented by the clustering of peptide masses [11-15] and the fact that most modification masses also are close to integers. In the mass range from 1 to 100 Da, approximately 75% of all integers have one or several modifications/substitutions with a mass close to it [6,7]. This means that at any random, but near-integer, distance from the true peptide m/z value, there is a considerable chance that one or several modifications will fit to this integer value. Furthermore, any positive near-integer value between 2 and 100 can be achieved by a combination of two modifications in the peptide. Thus, as the number of non-identified peptides increases, the likelihood of finding artifacts also increases.

In characterization high sequence coverage is desired, and one might therefore use as many peaks as possible, including low intensity peaks that would not have been used for identification purposes. Such peaks are more influenced by random noise, and are in general expected to have lower accuracy than high intensity peaks. Proteolytic cleavage specificity and efficiency are also not perfect. Thus, several factors will contribute to artifacts. A main strategy is to remove all peptides that can be identified with reasonable confidence before the initial mass shift comparison is performed. It is also recommended to search for peptides with unexpected cleavages or many missed cleavages by using MassSorter [2], FindPept [16] or similar tools, especially if an "unreliable" protease (like chymotrypsin) has been used.

Our primary objective with the algorithm is to promote the detailed low-throughput characterization of single proteins by indicating peptides that may contain modifications or substitutions. This can help in selecting peaks to target in fragmentation experiments. Furthermore, it is well known that a number of peptides are difficult to fragment in (LC-)MS/MS experiments. If an accurate instrument is used (e.g., Orbitrap or Q-TOF), it would be possible to extract suggestions for modifications from the survey scans, which could be the basis of alternative experiments (the use of other proteases, introduced chemical modifications, site-directed mutations in recombinant proteins, etc.).

## Competing interests

The authors declare that they have no competing interests.

## Authors' contributions

HB did the programming, in silico analyses, contributed ideas and was the main author of the manuscript. SOM made the initial description of the method, performed the mass spectrometry experiments, and participated in writing the manuscript. IE supervised the programming work, contributed ideas, and participated in writing the manuscript. All authors read and approved the final manuscript.

## Supplementary Material

Additional file 1 **Theoretical Examples**. A PDF file containing examples showing detection of modifications in an artificial dataset. Click here for file

Additional file 2 **Experimental Examples**. A PDF file containing details on MS experiments performed to show the proposed usage of the algorithm and software tool. Click here for file

Additional file 3 **Supplementary Material**. A PDF file containing additional information regarding a theoretical analysis of the degree of coverage and overlap between the proteases trypsin, chymotrypsin and gluC using 19,852 human proteins. Click here for file

## References

[B1] FindMod. http://ca.expasy.org/tools/findmod/.

[B2] Barsnes H, Mikalsen SO, Eidhammer I (2006). MassSorter: a tool for administrating and analyzing data from mass spectrometry experiments on proteins with known amino acid sequences. BMC bioinformatics.

[B3] MacCoss MJ, McDonald WH, Saraf A, Sadygov R, Clark JM, Tasto JJ, Gould KL, Wolters D, Washburn M, Weiss A (2002). Shotgun identification of protein modifications from protein complexes and lens tissue. Proc Natl Acad Sci USA.

[B4] Tsur D, Tanner S, Zandi E, Bafna V, Pevzner PA (2005). Identification of post-translational modifications by blind search of mass spectra. Nature Biotechnology.

[B5] Tanner S, Pevzner PA, Bafna V (2006). Unrestrictive identification of post-translational modifications through peptide mass spectrometry. Nat Protoc.

[B6] UniMod. http://www.unimod.org/.

[B7] Creasy DM, Cottrell JS (2004). UniMod: Protein modifications for mass spectrometry. Proteomics.

[B8] Java. http://www.java.com.

[B9] Cruciani V, Mikalsen SO (2007). Evolutionary selection pressure and family relationships among connexin genes. Biol Chem.

[B10] Cruciani V, Heintz KM, Husøy T, Hovig E, Warren DJ, Mikalsen SO (2004). The detection of hamster connexins: a comparison of expression profiles with wild-type mouse and the cancer-prone Min mouse. Cell Commun Adhes.

[B11] Gay S, Binz PA, Hochstrasser DF, Appel RD (2002). Peptide mass fingerprinting peak intensity prediction: extracting knowledge from spectra. Proteomics.

[B12] Wolski WE, Farrow M, Emde AK, Lehrach H, Lalowski M, Reinert K (2006). Analytical model of peptide mass cluster centres with applications. Proteome Sci.

[B13] Mann M Useful tables of possible and probable peptide masses. 43rd ASMS Conference on Mass Spectrometry and Allied Topics, Atlanta, GA.

[B14] Wool A, Smilansky Z (2002). Precalibration of matrix-assisted laser desorption/ionization-time of flight spectra for peptide mass fingerprinting. Proteomics.

[B15] Barsnes H, Eidhammer I, Cruciani V, Mikalsen SO (2008). Protease-dependent fractional mass and peptide properties. Eur J Mass Spectrom (Chichester, Eng).

[B16] FindPept. http://au.expasy.org/tools/findpept.html.

