# Supplementary material for: Blind search for post-translational modifications and amino acid substitutions using peptide mass fingerprints from two proteases
Source: BMC Res Notes. 2008 Dec 19;1:130. doi: 10.1186/1756-0500-1-130 (PMC2653020; doi:10.1186/1756-0500-1-130)
Supplement: Additional file 1 — Theoretical Examples. A PDF file containing examples showing detection of modifications in an artificial dataset. [file 1756-0500-1-130-S1.pdf]

Theoretical Examples

The tumor suppressor protein p53 (P04637) was used as a theoretical exercise for MassShiftFinder. Random inaccuracies (within ±25 ppm) were introduced in in-silico generated tryptic and chymotryptic peptides of p53. Some modifications and/or substitutions were then introduced in the peptides. The datasets were generated in Bergen and then sent to Oslo. The only information given was the identity of the protein and the protease used for each of the datasets. Information about the introduced modifications was not provided. Thus, the information available was rather similar to an experimental situation.

The data were first analyzed in MassSorter, and peptides fitting with theoretically unmodified peptides, peptides containing oxidized methionine and N-terminal pyroglutamic acid generated from glutamine were removed. In each of the test situations, five to seven peptides from each of the "digests" showed no obvious matches, and these peptides were transferred to MassShiftFinder. This dataset is referred to as filtered data.

We will here give some examples from one of the tests. Nine different combinations of peptides indicated a mass shift of -58.0 Da centered around the region 271-EVR-273. The mass shift immediately suggested a substitution, of which E271 to A would easily explain these data. However, also the double substitution E to G together with V to I/L would give the same mass shift. The former situation seemed the biologically most plausible, and was correct.

Another mass shift of 239.9 Da was detected for three pairs of overlapping peptides in the region 94-SSSVPSQK-101. This mass shift did not correspond to any single modification available in UniMod, and also not the sum of two modifications. Thus, this corresponded either to an unknown modification or more than two modifications. The region contained four S residues, and the mass shift was very close to three phosphorylations. Experimentally, this possibility could easily be tested by looking for unfocused post-source decay fragments in MALDI-TOF or neutral loss by MS/MS, or treating the sample with alkaline phosphatase.

The described example was also used to generate graphs describing how the number of suggested mass shifts changed as a function of the Mass Shift Accuracy, which was set at 0.2 Da during these exercises (TheoreticalExamples Fig. 1). The number of suggested mass shifts increased rapidly up to 0.1 (filtered data) or 0.2 Da (unfiltered data, where only m/z values corresponding to unmodified peptides were removed), and thereafter it plateaued until approx. 0.9 Da. Other theoretical and experimental examples followed similarly shaped curves. TheoreticalExamples Fig. 1 also indicates the advantage of removing peptides that can be identified with reasonable confidence before the analysis in MassShiftFinder.

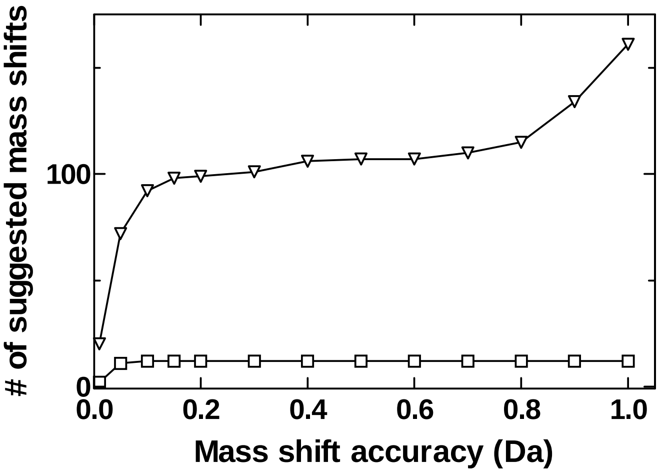

TheoreticalExamples Figure 1: Number of suggested hits by MassShiftFinder using artificially created digests and modifications in human p53. The generation of the datasets is described in the text above, and they were analyzed using the following settings: Peptide Accuracy, 25 ppm; Missed Cleavages, trypsin 1, chymotrypsin 2; Mass Shift Accuracy, variable (see figure); Mass Shift Threshold, 0.9 Da; Mass Shift Boundaries, -200 to 400, UniMod Accuracy, 0.1 Da, Peptide Mass Limits, 500 to 4000. Squares: peptides corresponding to unmodified p53 peptides, and peptides with oxidized methionines or with N-terminal pyroglutamic acid were removed before the analysis (filtered data). Triangles: only unmodified p53 peptides were removed before analysis (unfiltered data).
