# Supplementary material for: Blind search for post-translational modifications and amino acid substitutions using peptide mass fingerprints from two proteases
Source: BMC Res Notes. 2008 Dec 19;1:130. doi: 10.1186/1756-0500-1-130 (PMC2653020; doi:10.1186/1756-0500-1-130)
Supplement: Additional file 2 — Experimental Examples. A PDF file containing details on MS experiments performed to show the proposed usage of the algorithm and software tool. [file 1756-0500-1-130-S2.pdf]

Experimental Examples

Modifications in enolase: Tryptic and chymotryptic peptides from unmodified enolase were analyzed by MassShiftFinder. The same parameters were used as in the main text, except that mass shift boundaries were set to -20 to 200, missed cleavages were increased to 2 and post-translational and artefactual modifications (but no substitutions) were included in the search. For unmodified enolase, the areas 33-SIVPSGASTGVHEALEMR-50 and 369-GVMSHR-375 were both indicated to contain a modification with a mass shift of 16.0 Da. This would correspond to an oxidized M in both peptide sequences. A sodium adduct (mass shift of 22 Da relative to MH^+) was indicated for the enolase C-terminal peptides (the tryptic peptide 416-437 and chymotryptic peptide 421-437). A pair of m/z 2968.5 (tryptic) and 1745.9 (chymotryptic) was suggested as (K)289-RYPIVSIEDPFAEDDWEAWSHFK-311 and (W)308-SHFKTAGIQIVADDL-323 with a mass shift of -16 Da, corresponding to a β-elimination followed by reduction. This seemed rather unlikely, and the chymotryptic peptide, which had the more intense peak, was targeted for fragmentation. The fragment spectrum was consistent with the sequence 412-QLLRIEEELGDNAVF-426, and was thus cleaved C-terminal to N411. This serves as an example of an erroneous suggestion caused by an unexpected chymotryptic cleavage. (The tryptic digest of (unmodified) enolase had a coverage of 60%, the chymotryptic digest 62%.)

Modifications in ovalbumin: Again, several suggestions for oxidized methionines were obtained for positions 196, 210, 211, 222, 227 and/or 239. Another pair of peptides suggested potassium adducts occurring in the tryptic peptide 187-AFKDEDTQAMPFR-199 (m/z 1593.7) and the chymotryptic peptide 199-RVTEQESKPVQMMYQIGLF-217 (m/z 2322.2). Additionally suggested mass shifts will not be discussed as fragmentation was not productive, or the peaks were of very low intensity and fragmentation was not attempted. (The tryptic digest of ovalbumin had a coverage of 52%, the chymotryptic digest 48%.)

Guanidination of enolase: To test how MassShiftFinder would handle a massive amount of modifications, enolase was guanidinated. Enolase contains in total 14 arginines and 37 lysines. The majority of tryptic peptides (and numerous chymotryptic peptides) should therefore be modified by guanidination. Chemical modifications were included in the settings, but otherwise the same parameters as previously described were used. MassShiftFinder indicated a total of 183 tryptic-chymotryptic pairs that would give equal mass shifts when mapped to the enolase sequence. Among these, 48 were consistent with one or two guanidinations, i.e., mass shifts of 42 or 84 Da. Twenty-five of the pairs had K in the *Y* area, while 17 had K in both the *X* and *Z* (but not *Y*) area, and 6 had K in *X*, *Y* and *Z*. In several cases, more than one hit pointed to the same position(s). The pairs indicative of guanidination were removed. Further analysis of the remaining peaks using MassSorter [1] indicated that some of the remaining peptides were due to non-overlapping guanidinated peptides. These peptides were removed manually and the analysis was repeated, now giving a total of 32 hits, with 10 of the hits corresponding to one UniMod [2, 3] registered modification per peptide. Two of the UniMod hits could correspond to oxidations (mass shifts of 16 or 32 Da) of W57, M58, W273 and M287. Another hit with a mass shift of 100.1 Da was consistent with two guanidinations of Ks and one oxidation of M in each of the peptides 32-RSIVPSGASTGVHEALEMRDGDKSKW-57 (chymotryptic) and 57-WMGKGVLHAVK-67 (tryptic). Several of the peptides with no UniMod hits were subjected to TOF-TOF analysis, but in general these peaks were of low intensity. Only one of the

fragmentations was productive, a chymotryptic peak of m/z 1868.9 that paired with several tryptic peptides, giving mass shifts ranging from -19 to 175 Da, and pointing to several areas in enolase. The spectrum was resolved and corresponded to (F)152-LNVLN*GGSHAGGALALQEF-170, where N* (N156) had been deamidated, resulting in D, see ExperimentalExamples Figure 1. The non-modified peptide (at m/z 1867.9) was found in the enolase samples that had not been guanidinated. The NG combination is known to be particularly sensitive to deamidation [4, 5].

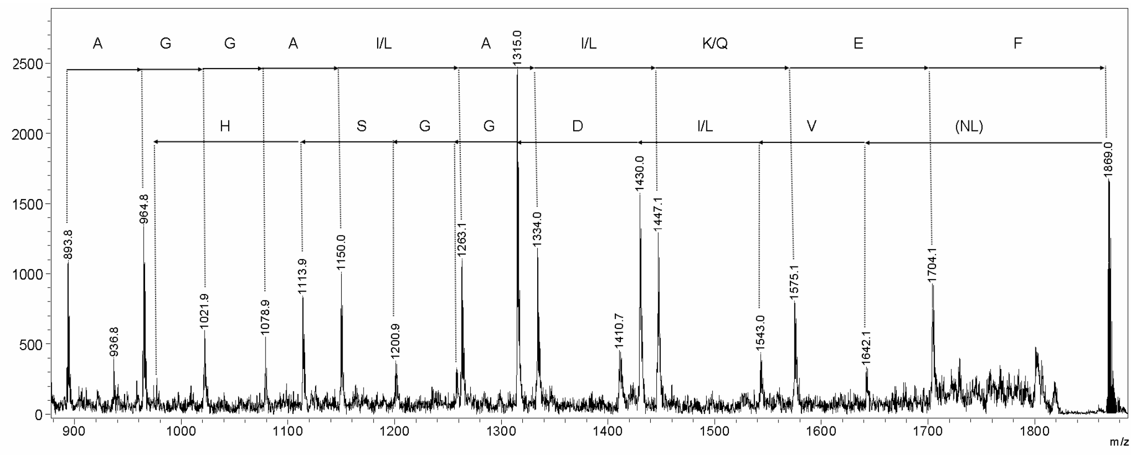

ExperimentalExamples Figure 1: Deamidation of Asn156 to Asp in guanidinated enolase. Partial fragmentation spectrum of the chymotryptic peptide, 152-LNVLNGGSHAGGALALQEF-170, from guanidinated enolase. The upper sequence is read from b-ions, and the lower from y-ions. The y-ions show the deamidation of N156 to D, changing the m/z value of the peptide from 1867.9 to 1868.9.

Methods

Materials: Chicken ovalbumin, enolase from Saccharomyces cerevisiae, anti-Cx43 antibody (C6219) and bovine chymotrypsin were bought from Sigma. Porcine trypsin was from Promega. Zip-Tips were obtained from Millipore.

Immunoprecipitation of connexin43: Connexin43 (Cx43) was immunoprecipitated from primary Syrian hamster embryo cells, Chinese hamster V79 cells and rat NRK52E cells by a polyclonal anti-Cx43 antibody. The separation of the immunoprecipitated proteins on gels, silver staining, band excision, destaining and dehydration with acetonitrile were performed essentially according to Gharahdaghi et al. [6]. The gel pieces were treated with trypsin or chymotrypsin overnight at 37 °C. The peptides were extracted by acetonitrile [6], dried down, and further desalted and purified by μC18-ZipTips. The samples were analyzed in a Bruker Ultraflex MALDI-TOF-TOF.

Preparation of enolase and ovalbumin samples: Enolase and ovalbumin were run on gels and the gel bands excised and further treated as described above, and digested with either trypsin or chymotrypsin. Enolase gel bands were also modified by guanidination before proteolytic treatment as described [7].

References

1. Barsnes H, Mikalsen SO, Eidhammer I: MassSorter: a tool for administrating and analyzing data from mass spectrometry experiments on proteins with known amino acid sequences. *BMC bioinformatics* 2006, 7:42.
2. UniMod [http://www.unimod.org/]
3. Creasy DM, Cottrell JS: UniMod: Protein modifications for mass spectrometry. *Proteomics* 2004, 4:1534 - 1536.
4. Krokhin OV, Antonovici M, Ens W, Wilkins JA, Standing KG: Deamidation of -Asn-Gly- sequences during sample preparation for proteomics: Consequences for MALDI and HPLC-MALDI analysis. *Anal Chem* 2006, 78:6645-6650.
5. Robinson AB, Rudd CJ: Deamidation of glutaminyl and asparaginyl residues in peptides and proteins. *Curr Top Cell Regul* 1974, 8:247-295.
6. Gharahdaghi F, Weinberg CR, Meager DA, Imai BS, Mische SM: Mass spectrometric identification of proteins from silver-stained polyacrylamide gel: A method for the removal of silver ions to enhance sensitivity. *Electrophoresis* 1999, 20:601-605.
7. Lindstad RI, Sylte I, Mikalsen SO, Seglen PO, Berg E, Winberg JO: Pancreatic trypsin activates human promatrix metalloproteinase-2. *J Mol Biol* 2005, 350:682-698.
