# Supplementary material for: Blind search for post-translational modifications and amino acid substitutions using peptide mass fingerprints from two proteases
Source: BMC Res Notes. 2008 Dec 19;1:130. doi: 10.1186/1756-0500-1-130 (PMC2653020; doi:10.1186/1756-0500-1-130)
Supplement: Additional file 3 — Supplementary Material. A PDF file containing additional information regarding a theoretical analysis of the degree of coverage and overlap between the proteases trypsin, chymotrypsin and gluC using 19,852 human proteins. [file 1756-0500-1-130-S3.pdf]

In Silico Analysis Of Overlapping Peptides

The described algorithm depends on the comparison of the digests from two proteases, and that the resulting peptides overlap. In turn, the degree of overlap depends mainly on the sequence coverage achieved. By applying the cleavage rules of a protease, along with peptide mass boundaries for mass spectrometry data and a maximum number of allowed missed cleavages, a theoretical upper boundary for sequence coverage can be determined for all protein-protease pairs. We therefore performed an analysis of all human proteins in the Swiss-Prot [1] database (Release 54, 19852 proteins) to investigate how different proteases theoretically affected the sequence coverage. All SwissProt proteins were in silico digested by trypsin (cleaves after R and K, unless followed by P), chymotrypsin (cleaves after F, Y, W and L, unless followed by P) and gluC (cleaves after E and D, unless followed by P). A maximum of one missed cleavage was allowed (two missed cleavages were also tested, but had little impact on the results). To emulate mass spectrometry conditions only peptides between 500 and 3500 Da were used.

The ionization and intensity of peptide peaks detected in MALDI instruments partly depend on the presence of certain amino acids, especially R [2], but also F, L and P [3]. Only peptides containing at least one of these amino acids were included in the analysis. By this restriction, 8.3% (trypsin) to 9.3% (gluC) of the peptides were removed, which on the average corresponded to 3 to 4 peptides per protein, see Supplementary Table 1. The theoretical sequence coverages for the single proteases are shown in Supplementary Table 2, and pairwise comparisons of the sequence coverages are plotted in Supplementary Figure 1. A further comparison of the theoretical sequence coverages for the three proteases, showed that chymotrypsin had higher (theoretical) coverage than trypsin in 49.7% of the proteins, and gluC in 44.6% of the cases, see Supplementary Table 3.

The same datasets were used to analyze how much the coverage for different proteases overlaps, see Supplementary Figure 2. For both protease pairs, trypsin vs. chymotrypsin and trypsin vs. gluC, around 50% or more of the proteins had a theoretical overlap higher than 50%.

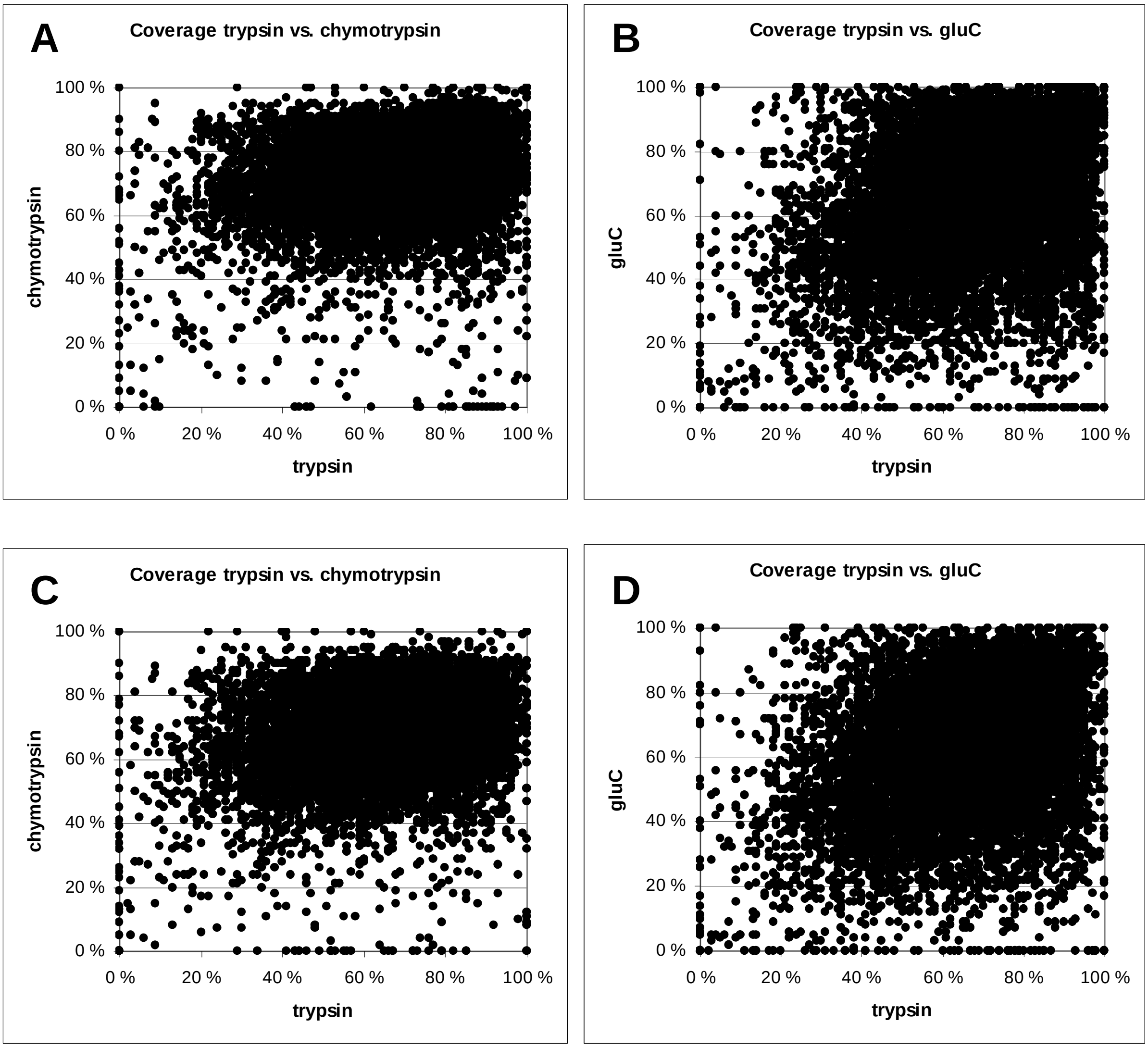

Supplementary Figure 1: Comparison of coverage degrees for 19,852 human proteins (Swiss-Prot November 22nd 2007) theoretically digested by trypsin, chymotrypsin and gluC. For **A** and **B** all peptides are used, while in **C** and **D** only peptides containing at least one R, F, L or P are included. Lower mass limit: 500, upper mass limit: 3500, maximum missed cleavages: 1. Chymotrypsin was used with the specificity FYWL.

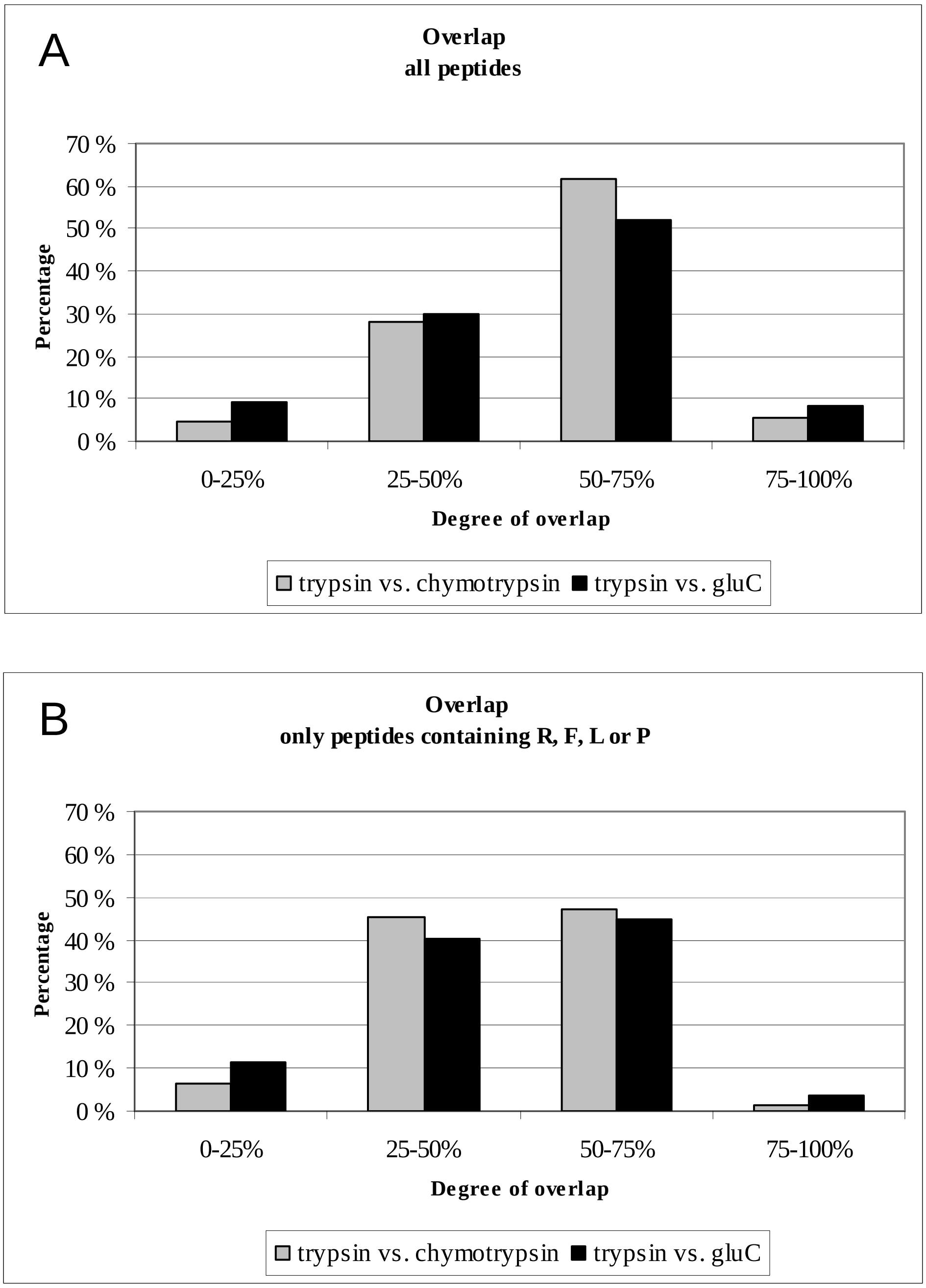

Supplementary Figure 2: An overview of the degree of overlap for 19,852 human proteins theoretically digested by trypsin, chymotrypsin and gluC. The degree of overlap is calculated as the percentage of the total sequence covered by both of the proteases. It is divided into four groups, and the number of proteins in each group is counted. In **A** all peptides are used, while in **B** only peptides containing at least one R, F, L, or P are included. Lower mass limit, 500; upper mass limit, 3500; maximum missed cleavages, 1; chymotrypsin cleaves after F, Y, W and L.

	Trypsin	Trypsin	Chymo-trypsin FYWL	Chymo-trypsin FYWL	Chymo-trypsin FYW	Chymo-trypsin FYW	GluC	GluC
**Coverage degree**	**All**	**RFLP**	**All**	**RFLP**	**All**	**RFLP**	**All**	**RFLP**
**0-25%**	1.6 %	1.8 %	0.9 %	1.1 %	4.9 %	5.5 %	2.5 %	3.0 %
**25-50%**	8.4 %	10.1 %	2.0 %	4.0 %	19.6 %	22.2 %	10.4 %	12.3 %
**50-75%**	40.0 %	50.5 %	38.5 %	61.0 %	44.5 %	47.2 %	39.9 %	50.5 %
**75-100%**	50.0 %	37.6 %	58.6 %	34.0 %	31.0 %	25.1 %	47.1 %	34.2 %

Supplementary Table 1: Theoretical coverage of 19,852 human proteins. Lower mass limit, 500; upper mass limit, 3500; maximum missed cleavages, 1. The percentage of proteins within each coverage group is given as all peptides (columns marked "All"), or only peptides containing at least one R, L, F, or P (columns marked "RFLP"). As an example, 50.0% of the proteins have a coverage degree between 75 to 100% when digested with trypsin, and this decreases to 37.6% if only peptides containing at least one R, L, F, or P are included.

	Average #peptides per protein	Total #peptides all proteins
**Trypsin All**	33.8	668266
**Trypsin RFLP**	31.0	612652
**Chymotrypsin All**	43.2	853327
**Chymotrypsin RFLP**	39.1	773232
**GluC All**	34.2	672133
**GluC RFLP**	30.9	607306

Supplementary Table 2: An overview of the number of of peptides with and without the constraint that all peptides have to include at least one R, F, L or P. Lower mass limit, 500; upper mass limit, 3500; maximum missed cleavages, 1; chymotrypsin cleaves after F, Y, W and L.

Trypsin (T) vs. chymotrypsin (C) all peptides

Average difference:	-2.9 %
Coverage T >= coverage C	46.2 %
Coverage T < coverage C	53.8 %

Trypsin (T) vs. gluC (G) all peptides

Average difference:	1.9 %
Coverage T >= coverage G	55.1 %
Coverage T < coverage G	44.9 %

Trypsin (T) vs. chymotrypsin (C) RFLP*

Average difference:	-1.2 %
Coverage T >= coverage C	50.4 %
Coverage T < coverage C	49.6 %

Trypsin (T) vs. gluC (G) RFLP*

Average difference:	2.0 %
Coverage T >= coverage G	55.4 %
Coverage T < coverage G	44.6 %

Supplementary Table 3: Comparison of the overall coverage degrees for 19852 human proteins theoretically digested by trypsin, chymotrypsin and gluC. Lower mass limit: 500, upper mass limit: 3500, maximum missed cleavages: 1. Chymotrypsin is used with the specificity FYWL.
*Only peptides containing R, F, L or P were included.

References

1. Swiss-Prot [http://au.expasy.org/sprot/]
2. Krause E, Wenschuh H, Jungblut PR: The dominance of arginine-containing peptides in MALDI-derived tryptic mass fingerprints of proteins. *Anal Chem* 1999, 71:4160-4165.
3. Baumgart S, Lindner Y, Kühne R, Oberemm A, Wenschuh H, Krause E: The contributions of specific amino acid side chains to signal intensities of peptides in matrix-assisted laser desorption/ionization mass spectrometry. *Rapid Commun Mass Spectrom* 2004, 18:863-868.
